# Biologically Modified Polyether Ether Ketone as Dental Implant Material

**DOI:** 10.3389/fbioe.2020.620537

**Published:** 2020-12-18

**Authors:** Zhangyu Ma, Xingyu Zhao, Jing Zhao, Zhilong Zhao, Qihui Wang, Congxiao Zhang

**Affiliations:** ^1^Department of Stomatology, The First Hospital of Jilin University, Changchun, China; ^2^Department of Bone and Joint Surgery, The First Hospital of Jilin University, Changchun, China; ^3^Department of Stomatology, China-Japan Friendship Hospital, Beijing, China

**Keywords:** Polyether ether ketone, surface modification, blending modification, biological activity, dental orthopedic implants

## Abstract

Polyether ether ketone (PEEK) is a non-toxic polymer with elastic modulus close to human bone. Compared with metal implants, PEEK has advantages such as evasion of stress shielding effect, easy processing, and similar color as teeth, among others. Therefore, it is an excellent substitute material for titanium dental orthopedic implants. However, PEEK’s biological inertia limits its use as an implant. To change PEEK’s biological inertia and increase its binding ability with bone tissue as an implant, researchers have explored a number of modification methods to enhance PEEK’s biological activities such as cellular compatibility, osteogenic activity, and antibacterial activity. This review summarizes current biological activity modification methods for PEEK, including surface modification and blending modification, and analyzes the advantages and disadvantages of each modification method. We believe that modified PEEK will be a promising dental and orthopedic implant material.

## Introduction

**FIGURE 1:**
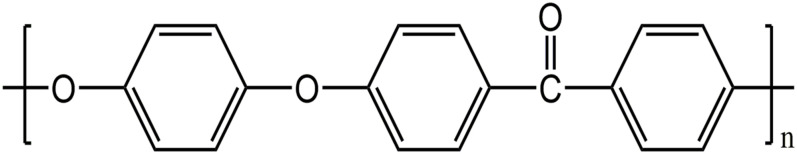
PEEK’s molecular structure.

Polyether ether ketone (PEEK) is a member of the polyaryletherketone family, which has an aromatic backbone combining the ketone and ether functional groups between the aryl rings. It is a high-performance engineering plastic with many excellent properties (Williams, 2008) such as chemical stability, excellent heat resistance and machinability, frictional resistance, good biocompatibility, elastic modulus close to human bone, and good X-ray penetration (Toth et al., 2006; Kurtz and Devine, 2007; Zhao et al., 2013; Wang et al., 2014; Abdullah et al., 2015; Figure 1). Owing to PEEK’s excellent mechanical properties, minimal immunotoxicity, and overall good processing performance (Toth et al., 2006; Kurtz and Devine, 2007; Zhao et al., 2013; Wang et al., 2014; Abdullah et al., 2015), it has become an alternative to metal implants and the preferred material for plastic and trauma surgery since the late 1990s (Kurtz and Devine, 2007). PEEK can overcome some of the limitations of metal implants such as stress shielding and metal allergy. PEEK is currently used as an orthopedic implant in clinical practice and has achieved good results (Song et al., 2010, 2011).

**FIGURE 2:**
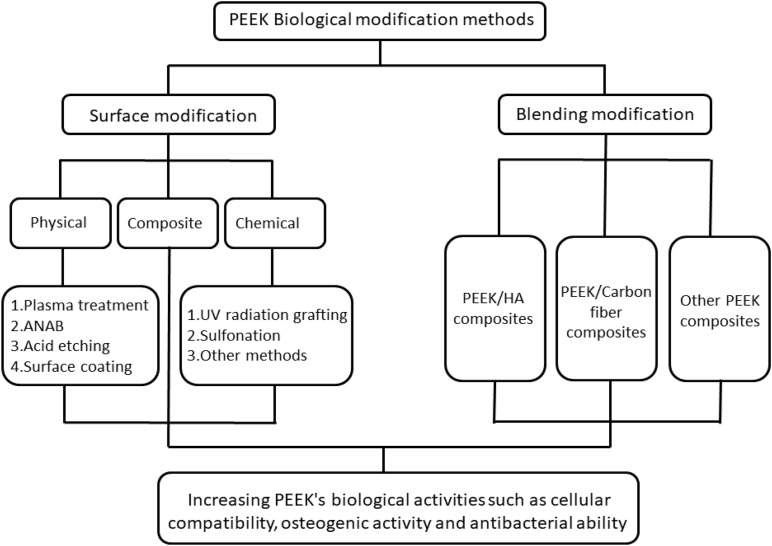
The classification of PEEK’s biological modification methods.

In recent years, based on several extensive PEEK research studies, its excellent performance has also attracted the attention of researchers in the field of stomatology. PEEK has similar mechanical properties to the hard tissue of the tooth and bone and its color is similar to that of the tooth, reflecting its great potential as a material for fixed prosthodontics (Ouyang et al., 2016). At present, there have been some research studies on PEEK as the restoration material for fixed-definition dental bridge brackets, implant abutments, and implants (Santing et al., 2012; Tannous et al., 2012; Najeeb et al., 2016). However, when used as an implant, PEEK has insufficient biological activity and cannot form good osseointegration with the surrounding bone tissue. These defects severely limit the practical clinical application of PEEK. Therefore, in recent years, various modification methods that can enhance PEEK’s biological activities such as cell compatibility, osteogenic activity, and antibacterial ability have become research hotspots. This paper reviews the biological modification methods of PEEK. The classification of PEEK modification is shown in Figure 2, which is mainly divided into surface modification and blending modification. Surface modification includes physical modification, chemical modification, and composite modification, which are a series of modification methods to improve PEEK’s biological activity by changing its surface morphology and active ingredients. Blending modification includes various modification methods to improve PEEK biological activity by mixing PEEK with hydroxyapatite (HA), carbonfiber (CF), zinc oxide (ZnO), and other bioactive substances to prepare composite materials. These modification methods will be reviewed in this paper.

## Surface Modification

As bone implant materials, the cytocompatibility on the surface of the materials is very important. The implant materials with good cytocompatibility can form good bone binding with the surrounding tissues after being implanted into the human body. The physical and chemical structure of the surface of bioactive materials can significantly affect the cytocompatibility of the surface. Generally, bioactive materials with rougher surface structure and better hydrophilicity are more conducive to the adhesion and proliferation of cells on the surface of the material, so that the material can form good bone binding with surrounding tissues. Surface modification methods are aimed at constructing biologically active structures or introducing biologically active substances on the PEEK surface by various means to increase the roughness and hydrophilicity of the material surface. These methods only act on PEEK surface without changing PEEK’s mechanical properties. According to the properties of modification methods, surface modification methods can be divided into three types: physical surface modification, chemical surface modification, and composite surface modification.

### Physical Surface Modification

This is a relatively simple method. Common physical modification methods include plasma treatment, neutral atomic beam technology, acid corrosion, and various surface coatings. These methods improve the biological activity of PEEK by constructing cell-based structures on the surface or by coating the surface with active substances.

#### Plasma Treatment

Plasma treatment refers to the use of N_2_, NH_3_, O_2_, Ar, H_2_, and other gas plasma treatment PEEK surface. This method improves the surface hydrophilicity, biological activity, and biocompatibility of PEEK by changing its surface morphology or forming functional groups with biological activity on PEEK surface. Novotna et al. (2015) modified PEEK surface by argon plasma and prepared samples with argon plasma processing time of 120, 240, and 480 s. After modification, the author systematically studied the changes of PEEK surface morphology before and after modification. The results showed that the roughness of the modified samples increased significantly, and as shown in Table 1, the hydrophilicity and oxygen content of the modified samples were significantly enhanced, while the rough surface, good hydrophilicity, and oxygen-containing active groups could promote the adhesion and growth of cells. The authors then introduced mouse fibroblasts and human osteoblasts on different sample surfaces to observe the growth of these two kinds of cells before and after modification. *In vitro* cell experiments showed that plasma treatment could significantly enhance cell adhesion, proliferation, and metabolic activities of the two cell lines. These results showed that the biological activity of PEEK surface was significantly improved by argon plasma treatment. The modified PEEK surface was more conducive to cell adhesion and proliferation as well as osteogenic differentiation, so it is easier to form a good bone binding with the surrounding tissues as an implant.

**TABLE 1:** Contact angle and X-ray photoelectron spectroscopy (XPS) elemental analysis results.

	WCA (°)		Element concentration (at%)	
Sample	15 min	14 days	C (1 s)	O (1 s)
PEEK	79.5 ± 2.4	79.5 ± 2.4	88.8	11.2
PEEK/120 s	23.2 ± 1.8	55.1 ± 2.2	67.3	32.7
PEEK/240 s	21.8 ± 1.3	48.3 ± 1.9	60.1	39.9
PEEK/480 s	18.9 ± 1.7	64.7 ± 2.1	51.4	48.6

In the study by Waser-Althaus et al. (2014), PEEK surface was treated with oxygen and ammonia plasma with a power of 10, 50, 100, and 200 W for 5 min; further, carboxylic acid, ester, and amine functional groups were introduced to increase the hydrophilicity of the material and change the surface morphology. Compared with pure PEEK, both plasma treatments could change PEEK surface morphology and increase PEEK hydrophilicity and protein adsorption capacity, but oxygen plasma treatment was significantly more effective than ammonia plasma treatment under the same conditions. These results show that the modification ability of different plasmas is different, and the surface structure of the sample can affect the hydrophilicity of the sample surface and the protein adsorption ability. The authors inoculated adipose tissue-derived mesenchymal stem cells (adMSCs) onto PEEK samples and conducted a series of cell experiments to evaluate the effect of plasma treatment on cell proliferation and differentiation. The cellular experiment results showed that compared with pure PEEK, treated PEEK significantly enhanced adhesion, proliferation, and osteogenic differentiation of adMSCs. These results indicated that plasma treatment can effectively enhance the biological activity of PEEK surface. These results showed that PEEK treated with oxygen and ammonia plasma could better bind to bone tissue, achieving the modification purpose of improving PEEK biological activity and promoting bone binding formation around the implant.

Surface modification methods for plasma treatment are repeatable, cheap, and simple to operate and can be applied to the modification of materials of various shapes and structures. However, the resultant modification effect lasts for a limited time, and the modified sample surface can only maintain the biological activity for a short period (Petlin et al., 2017). To increase the duration of the modification effect, researchers have been developing and studying new modification methods, among which the modification effect of the accelerated neutral atomic beam modification method can last for a long time and has less influence on the structure of the material.

#### Accelerated Neutral Atom Beam

Accelerated neutral atom beam (ANAB) technology is an ultra-shallow surface treatment technique that accelerates positively charged gas clusters by high electric potential, controlling the average energy of individual atoms between 10 eV per atom and 100 eV per atom. Because the energy carried by each atom is very small, the influence of these atoms on the structure of the material when they act on the surface is far less than that of other modification methods. The modification depth of the surface of the material is only 2–3 nm (Kirkpatrick et al., 2013; Ajami et al., 2017). Therefore, this modification method is favored by many researchers.

**FIGURE 3:**
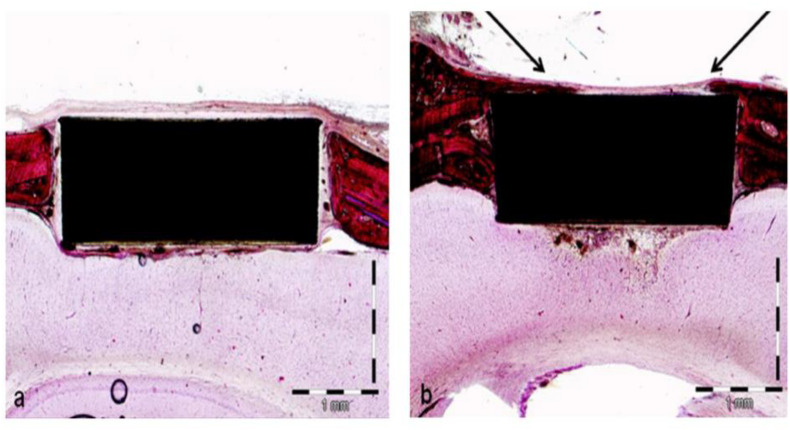
The tissue growth around unmodified pure PEEK sample **(a)** and ANAB-modified sample **(b)**.

Khoury et al. (2013) used an intense beam of cluster-like packets of accelerated unbonded neutral argon gas atoms to modify PEEK and conducted a series of cellular and animal-model experiments. The surface roughness and hydrophilicity of the modified samples increased with ANAB technology. Human fetal osteoblast cells (hFOBs) were inoculated on PEEK surface before and after modification and on the surface of titanium alloy in cell experiments. The results showed that the cell proliferation of cells on modified PEEK surface was significantly better than that of cells on unmodified PEEK surface, and the number of modified PEEK surface cells was similar to that of titanium alloy, the most commonly used clinical implant material. To further explore the biological activity of the modified samples, the authors conducted *in vivo* experiments to prepare the critical skull defect model of rats, and implanted the PEEK samples, before and after modification, into the defect for 4 weeks to observe the binding of the samples to the surrounding tissues. The results of animal experiments are shown in Figure 3. There was only a small amount of fibrous tissue around the unmodified pure PEEK sample, while the bone tissue around the ANAB modified sample was significantly increased. These results confirmed that ANAB technology can enhance PEEK biological activity by changing PEEK surface topography and increasing roughness, which is conducive to the formation of binding of PEEK implants to surrounding tissue and bone.

In another study by Khoury et al. (2017), ANAB technology was used to modify PEEK. In this experiment, the number of surfactant groups in each sample group was measured, and the results showed that the number of surface-active groups in the sample group modified by ANAB method was significantly higher than that of the unmodified sample group and the plasma-treated sample group. These results proved that both ANAB modification and plasma modification could increase PEEK bioactivity by forming hydroxyl and carboxyl groups on PEEK surface; the modification effect of ANAB technology was better than that of plasma treatment. Subsequent *in vitro* biological experiments and animal experiments showed that ANAB-technology modified samples were more conducive to mineral deposition, effectively improved the ability of cell adhesion and proliferation, and the expression of genes related to cell osteogenesis; they also formed good bone binding in animals. These results indicated that ANAB technology can effectively improve PEEK biological activity, which may be enhanced by introducing various active groups on PEEK surface.

These studies have proved that ANAB technology can increase PEEK surface roughness by changing PEEK surface morphology. Introduction of the hydroxyl, carboxyl, and other active groups on PEEK surface could further effectively improve PEEK’s cellular compatibility and bone binding ability. Compared with the gas plasma surface modification technology, the controllable operation of ANAB on the surface of materials is between 2 and 3 nm (Ajami et al., 2017), which has little influence on the structure of PEEK and has long-term stability. However, the cost is high and the operation is complex.

#### Acid Etching

**FIGURE 4:**
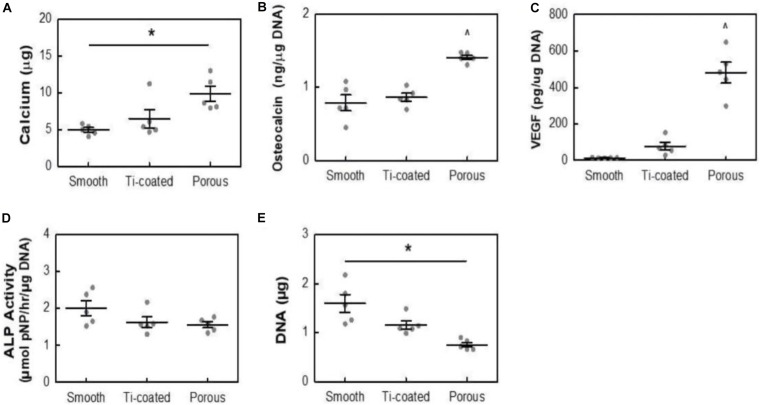
Calcium content **(A)**, osteocalcin **(B)**, VEGF **(C)**, ALP Activity **(D)**, and DNA content **(E)** of MC3T3 cultures in osteogenic media at 14 days. All data are from identical sample wells, except for calcium, which was from parallel cultures. *p < 0.05, ^*p* < 0.05 vs. other groups (two-way ANOVA, Tukey’s test, *n* = 5). Mean ± SE.

A previous study (Wong et al., 2009; Wang et al., 2019) showed that the porousstructure on the surface of bone implants is conducive to the formation of bone binding. Acid etching surface modification method refers to the corrosion of PEEK with strong acid such as concentrated sulfuric acid, and the preparation of porous structure conducive to cell growth on PEEK surface to enhance PEEK biological activity and facilitate the modification of PEEK surface bone binding. Therefore, Torstrick et al. (2018) adopted acid etching to form a porous structure on PEEK surface. The pore morphometrics data are shown in Table 2. The data showed that the pores were uniform in size and intercorrelated internally. To study the effect of porous structure on PEEK biological activity, MC3T3 cells were simultaneously inoculated on smooth unmodified PEEK, porous PEEK, and plasma-sprayed, titanium-coated PEEK. After a 14-day culture, calcium content, osteocalcin content, vascular endothelial growth factor (VEGF) secretion, alkaline phosphatase activity, and DNA content were measured. The results are shown in Figure 4. Increased calcium and osteocalcin content and decreased DNA content of cells on porous PEEK surface represent increased cell-mediated mineralization and enhanced osteogenic differentiation activity of the cells on the porous PEEK surface. However, the increased secretion of vascular endothelial influencing factor may be the result of lack of oxygen in the deep pores. Alkaline phosphatase is a marker of early osteogenic differentiation, so there was no significant difference in alkaline phosphatase activity between different sample groups at 14 days. To further investigate the *in vivo* osseointegration ability of porous PEEK, the authors implanted different samples into the proximal tibia of rats, and observed the bone binding of each group 8 weeks later by computed tomography (CT), histological analysis, and pull-out tests. The results indicated that the osseointegration ability with surrounding tissues of the three samples showed the trend in the order of porous PEEK > titanium-coated PEEK > smooth pure PEEK implants, with porous PEEK implants requiring the greatest extractive force than the other two samples. These results indicate that the porous structure can effectively improve PEEK’s bone-binding ability, and the porous structure can intermesh with the bone, providing better bone binding strength *in vivo*.

**TABLE 2:** Porous PEEK pore morphometrics.

	Porous PEEK
Porosity (%)	68.7 ± 0.5
Pore size (μm)	340 ± 8
Strut spacing (μm)	244 ± 2
Strut thickness (μm)	114 ± 2
Pore depth (μm)	523 ± 17
Inter-connectivity (%)	99.96 ± 0.01

*Mean ± SE for all values.*

**FIGURE 5:**
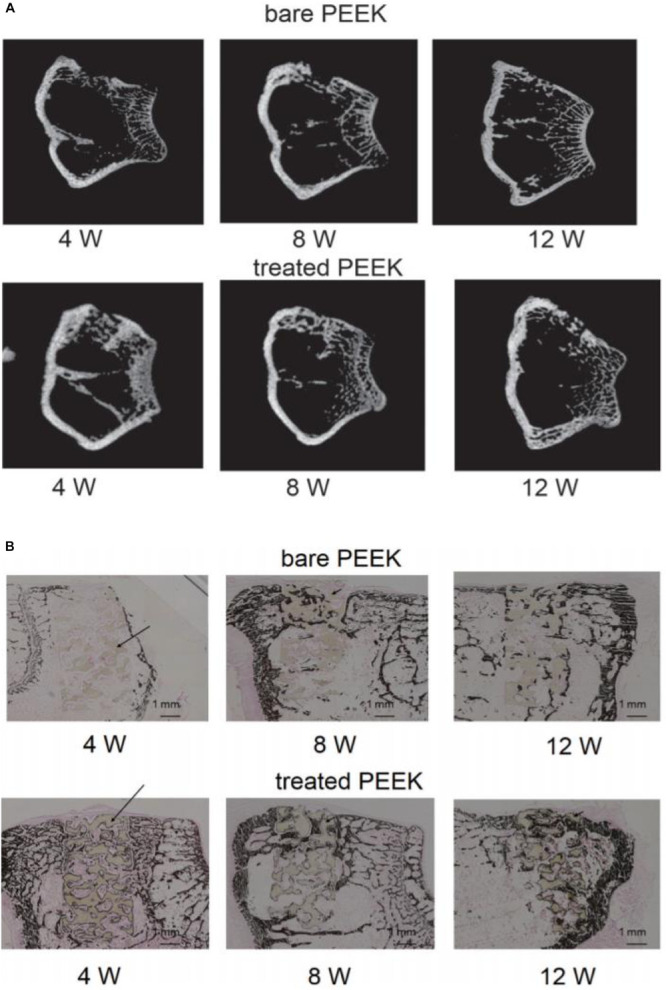
Micro computed tomography (micro-CT) images **(A)** and von Kossa staining images **(B)** at 4, 8, and 12 weeks after surgery. Dark brown staining represents bone tissue and black arrows show the PEEK materials in the bone defect.

Hieda et al. (2017) treated the PEEK scaffold with 70% porosity with concentrated sulfuric acid and potassium carbonate solution successively, formed a porous foam structure on the surface of the scaffold, and then implanted the scaffold material into the femur of rabbits. At the time points of 4, 8, and 12 weeks, respectively, they studied the bone binding of different samples *in vivo* through micro-CT and various staining methods. The results of CT and von Kossa staining are shown in Figure 5. The results showed that mature bone tissue had grown in the modified scaffold sample after 4 weeks, while only a small amount of bone tissue appeared around the scaffold in the unmodified sample. At 8 and 12 weeks, mature bone grafts also appeared in the unmodified scaffold; however, the area of bone tissue was lower than that in the modified PEEK scaffold. These results indicate that the porous structure can effectively improve the osteogenic activity of PEEK implants. In general, the acid etching modification method is simple to operate, but PEEK’s structure is destroyed during the modification, which affects its excellent mechanical properties.

#### Surface Coating

In addition to plasma treatment, neutral atom beam technique, and acid treatment to improve PEEK bioactivity by introducing active groups or changing surface morphology, researchers have also adopted many different methods to prepare bioactive coatings on PEEK surface to achieve modification. The literature on modification of PEEK surface coating is summarized in Table 3.

**TABLE 3:** Examples of surface coating modification literature.

Coating material	Method	Modification effect	Advantages	Disadvantages	Authors
Ti	Plasma-spray	Enhances osteogenic activity	Simple operation and low cost	Insufficient bonding strength of coating and high temperature may damage PEEK’s original structure (Stiles-Shields et al., 2015)	Hwang et al., 2015
Ti	Electron beam deposition	Enhances proliferation and osteogenic differentiation of human mesenchymal stromal cells and Mc3t3-e1	High coating accuracy	Low efficiency and high cost	Han et al., 2010; Elschner et al., 2015
TiO_2_	High-power pulsed magnetron sputtering	Enhances cytocompatibility of PEEK surface	The coating is more stable and has better mechanical properties	PEEK surface structure is damaged during modification	Yang et al., 2015
TiO_2_	Sol-gel method	Enhances ability of bone integration	No damage to PEEK surface structure	Long reaction time and high cost	Shimizu et al., 2016; Shimizu et al., 2017
TiO_2_	Arc ion plating	Enhances osteogenic properties	Simple operation and low cost	The coating is not uniform and has large particles	Tsou et al., 2015
HA	Spin coating	Enhances the ability of bone integration	Low cost and uniform coating	Limited preparation efficiency	Barkarmo et al., 2013
HA	Cold Spraying	Enhances the proliferation and osteogenic differentiation of human bone marrow mesenchymal stem cells	The bonding strength of the coating is better	The cost is high and the operation is dangerous	Lee et al., 2013
Nano ZrP	Spin coating	Enhances the ability of bone integration	Low cost and uniform coating	Limited preparation efficiency	Kjellin et al., 2020
Nano Si_3_N_4_	Suspension coating and melt bonding	Enhances the ability of cytocompatibility and bone integration	Excellent modification effect	Complex modification steps	Dai et al., 2019

### Chemical Surface Modification Methods

Chemical surface modification mainly refers to the chemical reaction between PEEK and various biologically active substances, and the introduction of biologically active substances or groups on PEEK surface in the form of various chemical bonds, in order to achieve the purpose of modification. The binding strength between PEEK and the active substance bound by chemical bonds is better than the physical method, and there are many biologically active substances to choose from.

#### Ultraviolet Radiation Grafting

Ultraviolet (UV) radiation grafting is a simple chemical modification method. UV irradiation can break the carbonyl chemical bonds in PEEK’s main chain structure and generate free radicals, which can induce polymerization of bioactive olefins and other monomers, thus grafting various bioactive substances onto the PEEK surface. This method is relatively simple to operate and has low cost of irradiation equipment. Besides, this modification does not affect the structure and properties of the material, and there is a chemical bond between the active substance and PEEK, which is stronger than the simple physical bond in physical modification. More active substances can be selected for chemical modification than the simple physical modification. Therefore, many researchers introduced various active substances into PEEK’s surface by this method and explored the changes of PEEK biological activity before and after modification.

**FIGURE 6:**
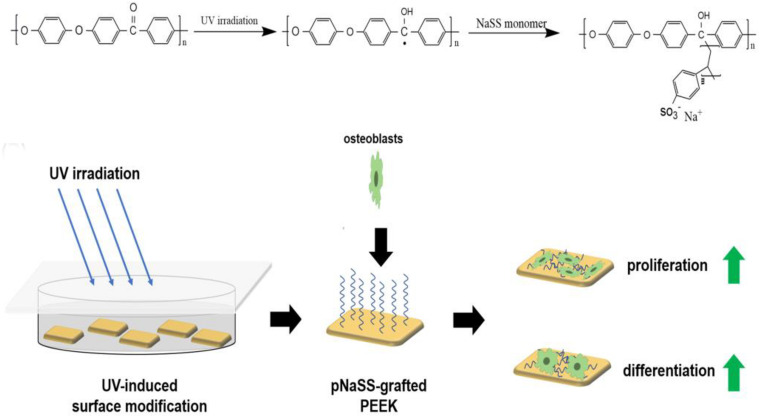
Preparation route and *in vitro* evaluations of pNaSS-grafted PEEK.

Ma Z. et al. (2020) grafted sodium polystyrene sulfonate onto PEEK surface by UV-induced grafting, thereby achieving the purpose of improving PEEK biological activity. The specific experimental procedure is shown in Figure 6. By controlling the UV exposure time, the modified samples were prepared with the grafting amount ranging from 0.59 ± 0.07 to 5.08 ± 0.21 mmol/cm^2^. Characterization tests showed that sodium polystyrene sulfonate was successfully grafted on PEEK surface and distributed evenly. *In vitro*-simulated mineralization test results showed that the mineral deposition capacity of PEEK surface was significantly improved after modification, and the Ca/P ratio of the deposited mineral was very close to that of calcium phosphate in human bone. The *in vitro* experiments also showed that the proliferation, spread, extracellular matrix secretion, calcification, alkaline phosphatase activity, and osteogene-related gene expression of modified PEEK surface cells were stronger than those of unmodified PEEK cells, and this trend increased with the graft amount. These results indicate that sodium polystyrene sulfonate grafted to PEEK surface by UV irradiation can effectively improve PEEK osteogenic activity, which is a positive significance for the clinical application of PEEK as an implant.

Owing to the complexity of the *in vivo* environment, simple *in vitro* cell experiments could not fully reflect the osseo-binding effect of the implant *in vivo*. Thus, after the phosphate group was grafted onto the PEEK surface by UV-induced one-step method, Zheng et al. (2019) conducted not just *in vitro* experiments but also animal experiments to further investigate the osseointegration of PEEK implant in animals. Characterization tests proved that UV irradiation successfully grafts phosphate groups to the PEEK surface without changing PEEK surface morphology and structure, effectively increasing PEEK surface hydrophilicity. The *in vitro* experiment results showed that the adhesion, proliferation, extracellular matrix mineralization, activity of osteogenic enzymes, and content of osteogenic-related genes were significantly increased in cells grown on the modified PEEK surface compared to those grown on the unmodified PEEK surface. The rabbit proximal tibia defect model was used to evaluate the *in vivo* biological activity of a different sample set of PEEK. In rabbits with tibial implants, the histological results after 12 weeks showed that the modified PEEK around the implant bone formation was significantly higher than that of the unmodified implant. The statistical analysis found that the modified sample of bone–implant contact ratio can reach 45%, the bone–implant contact ratio of the unmodified sample ratio was only 25%. These results indicate that this modification method can improve PEEK’s biological activity simply and effectively.

In addition to sodium polystyrene sulfonate and phosphate groups, a number of active substances are grafted onto PEEK surfaces. Zhao et al. (2017b) and Yousaf et al. (2014) also grafted polyacrylic acid, polyvinyl phosphonic acid, polybutyl acrylate, and other polymers on PEEK surface through UV irradiation. The results of these studies all showed that PEEK’s biological activity was significantly enhanced and the osteogenic capacity was improved after the introduction of the active substance. It shows that this modification method is feasible and effective.

#### Sulfonation Treatment

Sulfonated surface modification is a modification method that forms a porous structure on the surface of PEEK by concentrated sulfuric acid treatment or other methods and introduces a sulfonic acid group to enhance the biological activity of PEEK. The traditional sulfonation method directly treats PEEK with concentrated sulfuric acid solution to achieve sulfonation effect. Wan et al. (2020) recently discovered a new sulfonation method, which uses sulfur trioxide fumigation PEEK to achieve sulfonation. The effect of fumigation and sulfonation on PEEK structure is much less than that of traditional concentrated sulfuric acid sulfonation.

Zhao et al. (2013) sulfonated PEEK with traditional sulfonation method. According to different subsequent treatment methods, the modified samples were divided into high-sulfur samples treated with water and low-sulfur samples treated with water and acetone. The results of *in vitro* and animal experiments showed that the sulfonated samples with low sulfur concentration had stronger ability of cell proliferation, differentiation, and bone integration *in vivo* than the sulfonated samples with high sulfur concentration. Ouyang et al. (2016) also sulfonated PEEK using traditional methods, and obtained samples with different sulfur content after heat treatment under different conditions. For a dental implant, excellent antibacterial performance is essential in addition to good osteogenic activity. Therefore, the author not only conducted cytology test, but also conducted antibacterial experiment, and found that the modified samples can promote the proliferation and osteogenesis of mesenchymal stem cells of differentiation, and had a good resistance to *Staphylococcus aureus* and *Escherichia coli*. They then explored the effect of sulfur content on this modification. The results showed that, with the increase of sulfur content, the antibacterial activity of the samples increased, but the osteogenic ability decreased. Subsequently, the authors conducted animal experiments using a mouse femur defect model, and the result trend was consistent with the results of cell and antibacterial experiments *in vitro*. These results showed that sulfonated samples had good osteogenic ability and antibacterial property, but the release of sulfur from high-sulfur samples may lead to low pH around implants to inhibit the growth of cells to some extent, so it is very important to control the sulfur content of the modified samples to achieve the optimum osteogenesis and antibacterial ability.

**FIGURE 7:**
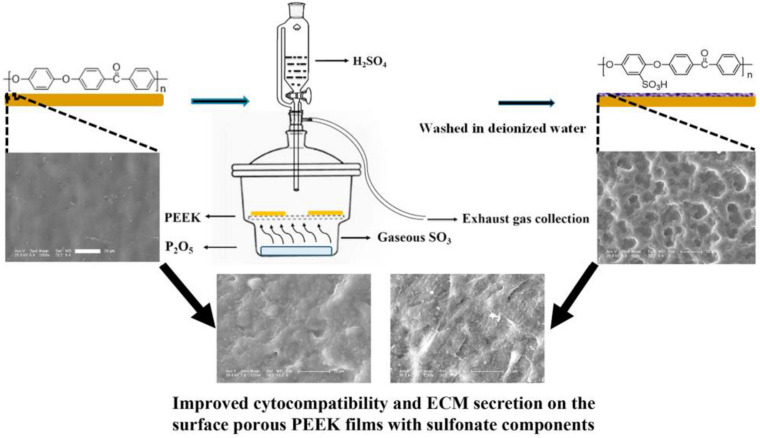
Schematic diagram of fabricating surface porous PEEK implants by gaseous SO_3_-induced controllable sulfonation.

In addition to the traditional sulfonation modification concentrated sulfuric acid, Wan et al. (2020) recently adopted a new gas fumigation method for sulfonation of PEEK. This method adopts gas fumigation of PEEK to form a microporous structure on its surface, and introduces a sulfonic group to obtain sulfonated PEEK with different sulfur content by controlling the fumigation time. Compared with the traditional method, this method can achieve sulfonation without destroying the structure of PEEK. The specific experimental process is shown in Figure 7. The fumigated sulfonated PEEK surface has uniform pores between 6 and 14 μm, and the modified material has good hydrophilicity, protein adsorption, and mineralization abilities, as well as good cytocompatibility, which can effectively promote cell adhesion, proliferation, and differentiation. These results indicate that the pore structure formed by fumigation on PEEK surface is significantly smaller and more uniform than that formed by conventional sulfonation methods. Moreover, this modification method effectively improves PEEK’s biological activity, and is a modified sulfonation method, which is worthy of further exploration regarding the effect of this method on PEEK’s antibacterial ability and biological activity *in vivo*.

The operation steps of the sulfonated surface modification method are relatively simple, and can form a porous structure conducive to cell growth on the PEEK surface, introduce sulfonic acid groups with biological activity, and improve PEEK’s antibacterial and osteogenic capabilities. However, the concentrated sulfuric acid and other modified reagents are dangerous to handle, so the experiment has certain limitations. Moreover, the traditional sulfonation modification method can destroy PEEK’s structure and inhibit the growth of PEEK surface cells when the sample surface contains too much sulfur.

#### Other Chemical Modification Methods

In addition to the ultraviolet irradiation graft and sulfonation modification, the researchers also adopted a variety of chemical substances to undergo reduction and hydrolysis reactions with PEEK, and introduced carboxyl, amino, and other active tubular groups on PEEK surface to achieve modification.

Zheng et al. (2015) reduced the carbonyl group on the surface of PEEK to a hydroxyl group, and then treated the hydroxylated pretreated PEEK sample with organosilane solution to form the functional surface layer of carboxyl group (–COOH). *In vitro* cell experiments showed that compared with hydroxylated PEEK and unmodified pure PEEK, carboxyl introduced on the surface of PEEK samples could better promote cell adhesion, spread, and proliferation. These results indicated that the introduction of carboxyl groups effectively enhanced the biological activity of PEEK surface.

Mahjoubi et al. (2017) introduced phosphate groups to the PEEK surface through a two-step diazo chemistry method. The modified samples had good hydrophilicity and mineral deposition ability. Further, the author tested the adhesion strength of the HA deposited on the surface of the sample with means of a scratch test. The results showed that the adhesion strength of the HA deposited on the surface of the modified sample with phosphate was stronger than the HA deposited on the unmodified PEEK surface. *In vitro* experiments showed that the samples with phosphate groups introduced on the surface could significantly improve the metabolic activity, and extracellular matrix mineralization of MC3T3-E1 cells. Animal experimental results showed that 3 months after modified PEEK implantation into rat skulls, the unmodified PEEK implant was surrounded by fibrous tissue, while there was no fibrous tissue around the modified PEEK implant and no evidence of mineral deposition, indicating that the introduction of phosphate groups could significantly enhance the osseointegration ability of PEEK implant.

**FIGURE 8:**
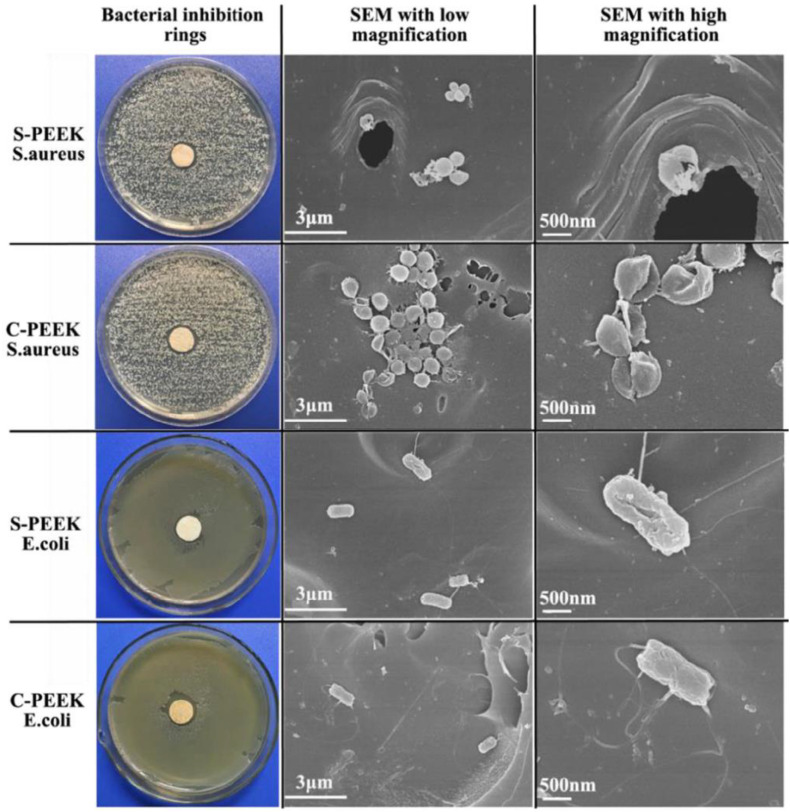
The bacterial inhibition rings and SEM images with low and high magnifications.

Infection is a common complication of implantation; hence, as a bone implant material, in addition to good bone-binding ability, antibacterial ability is also indispensable. He M. et al. (2019) chlorinated PEEK and then grafted two types of quaternary ammonium salts on PEEK surface through chemical reaction to obtain S-PEEK and C-PEEK samples modified by quaternary ammonium salt. Fourier transform infrared analysis, contact angle, and other characterization results proved that the two ammonium salts were successfully grafted onto PEEK surface and significantly improved its hydrophilicity. The antibacterial activity of the modified samples was evaluated by bacteriostatic circle test and plate colony-counting methods. As shown in Figure 8, obvious bacteriostatic rings appeared around the two modified samples, and the morphology of bacteria at high magnification showed that the surface of *E. coli* and *S. aureus* in contact with the modified samples was damaged, which proved that the antibacterial mechanism of quaternary ammonium salt was by contact sterilization. The results of plate colony counting showed that the antibacterial rates of the two modified samples against *S. aureus* and *E. coli* were >98 and >48%, respectively, which proved that the quaternary ammonium salt-modified PEEK could effectively improve the antibacterial ability of PEEK.

Such modification methods have the advantages of simple operation and low cost. However, given PEEK’s stable property, it is difficult to react with other substances, so the modification methods available at present are very limited. However, after the introduction of active groups on the surface, PEEK surface reactivity increases, making it easier to introduce other bioactive substances on PEEK’s surface, which is also one of the advantages of this surface modification method.

### Composite Surface Modification Methods

The human body environment is very complex. As an intramedullary implant material, PEEK’s single physical or chemical modification sometimes fails to meet clinical needs. Therefore, based on the original single physical or chemical modification, researchers adopted other modification methods to further introduce more active substances to more effectively improve PEEK’s biological activity and further improve the binding ability between the implant and surrounding tissues. This kind of surface modification method is the composite surface modification method. Wiacek et al. (2016a, b) introduced a coating of chitosan with antibacterial properties and good biological activity onto the surface of nitrogen-plasma-modified PEEK and tested the physicochemical properties and biological activities of the modified material. Experimental results showed that after nitrogen plasma treatment at low temperature, the surface roughness, free energy, and adhesion of the modified samples were obviously improved, and the adhesion stability of the chitosan coating on the PEEK surface could be greatly improved. Therefore, the modified PEEK surface could ensure a stable chitosan coating. The results of the contact angle and mechanical properties showed that the mechanical properties of PEEK did not change before and after the composite modification, while the hydrophilicity was significantly improved.

Pure porous structure can only increase PEEK’s biological activity to a limited extent. Therefore, some researchers choose to introduce more active substances on PEEK’s surface after acid modification to obtain a better modification effect. Sun et al. (2018) treated PEEK with concentrated sulfuric acid to create a porous structure on the PEEK surface, then introduced different concentrations of bone morphogenetic proteins-2 (BMP-2) into the porous sample surface. The samples were divided into BMP2-25, BMP2-50, and BMP2-100 groups according to different concentrations of BMP-2 solution added on each PEEK sample surface. Various characterization results showed that BMP-2 was successfully fixed in PEEK’s porous structure, and the hydrophilicity of the loaded BMP-2 protein sample was significantly enhanced and increased with the protein content. The release test results of BMP-2 protein showed that the release time of high-content samples was up to 28 days. The *in vitro* experimental results showed that the proliferation, adhesion, collagen secretion, alkaline phosphatase activity, and extracellular matrix calcification of the loaded protein samples were stronger than those of the control group. In this study, the author further introduced BMP-2, an active substance promoting osteoblastic differentiation of cells, into the sulfonated PEEK surface, which further enhanced the biological activity of the PEEK surface, reduced the adverse effect of sulfonated modified samples on cell growth and obtained better modification effect than simple sulfonated modification.

In addition to BMP-2, other active substances have also been introduced into the porous PEEK surface for better biological activity. Wan et al. (2019) also pretreated PEEK with concentrated sulfuric acid and prepared a porous structure conducive to bone integration on the material surface. Two growth factors—IGF-1 and BMP-2—were fixed on the porous surface of PEEK material through a polydopamine (pDA) coating to construct a biologically active PEEK implant material. Characterization tests showed that PEEK surface showed homogeneous pores with pore size between 0.24 and 0.74 μm after sulfuric acid treatment. After dopamine fixation, the hydrophilicity and protein adsorption ability of the sample surface were significantly enhanced. Cell experiments showed that after the two growth factors were fixed on the PEEK surface, the adhesion, proliferation, and spread of the growing cells on the PEEK surface became better, and the activity of osteoblast-related enzyme alkaline phosphatase and cellular matrix calcification also increased. Chen et al. (2012) and Sun et al. (2012) induced graft copolymerization of polyacrylic acid onto PEEK surface by UV irradiation to introduce carboxyl groups, then fixed bovine serum albumin (BSA) on the PEEK surface after graft activation. Experimental results showed that the biological activity of the modified PEEK surface was significantly improved.

**FIGURE 9:**
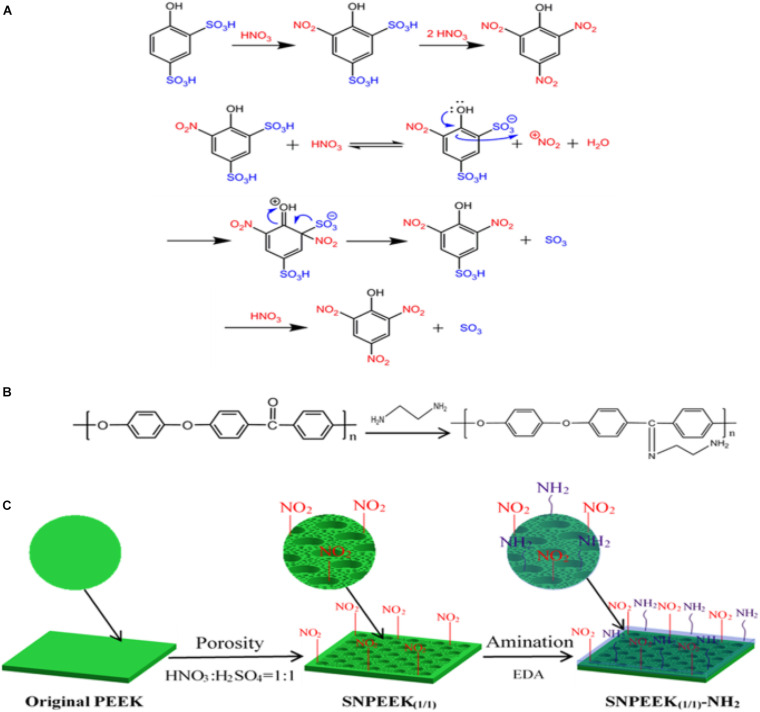
**(A)** Taking the preparation of picric acid from 4-hydroxybenzene-1, 3-disulfonic acid as an example, the mechanism of substituting the sulfonic acid group with nitrite for PEEK modification was expounded. **(B)** Grafting reaction between the EDA and ketone carbonyl group of PEEK. **(C)** Schematic diagram for the modification process of the PEEK surface.

As dental implant material, in addition to good cellular compatibility, good antibacterial ability is also essential. Ding et al. (2019) selected nitric acid and sulfuric acid in a ratio of 1:1 to treat PEEK surface, and constructed a three-dimensional porous structure conducive to bone growth on the PEEK surface. Then, chemical grafting was used to graft ethylenediamine, which has antibacterial activity, on the porous PEEK surface. The specific reaction mechanism is shown in Figure 9. Scanning electron microscopy, atomic force microscope, energy-dispersive spectroscopy, contact angle, and other characterization results showed that the amino group was successfully grafted onto porous PEEK surface, and the pores of the sample surface were evenly distributed, and the hydrophilicity was obviously enhanced. *In vitro* antibacterial tests and cytological testing showed that the composite modified samples showed excellent resistance ability to *E. coli* and *S. aureus*, and the modified samples had good cytocompatibility and osteogenic activity The rabbit femoral defect model was used in animal experiments, and the osseointegration of implants in different sample groups was evaluated by push-out tests and histological examination. The results showed that the composite modified samples were most closely bound to the surrounding tissues and required the greatest extrudability. Histological staining results at 1 month of implantation also showed no inflammatory response around the composite modified sample and the newest bone formation. These results proved that this modification method combines the advantages of the two methods and achieves better modification effect.

**FIGURE 10:**
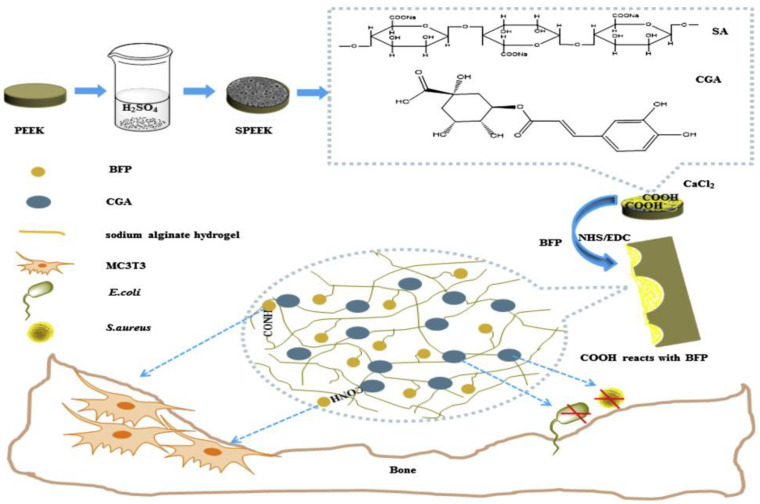
A preparation schematic of the drug-loaded/grafted peptide sodium alginate hydrogel bridging system, that endows SPEEK with dual biological applications: osteogenesis and antibacterial activity.

By combining sulfonation modification and surface coating modification, He X. et al. (2019) constructed a porous structure by sulfonation treatment on PEEK surface, and then introduced the hydrogel drug loading system of chlorogenic acid (CGA)/bone-induced growth factorinto the porous PEEK surface to further improve PEEK’s biological activity and antibacterial ability. The specific modification process is shown in Figure 10. The antibacterial experiment and *in vitro* cytological test results showed that the compound modification effectively improved PEEK’s antibacterial ability and promoted the proliferation, adhesion, and osteogenic differentiation of cells growing on the PEEK surface. Kuroda et al. (2018) treated PEEK by sulfuric acid etching and ultraviolet irradiation, and the experimental results showed that modified PEEK’s bone binding ability was improved and could inhibit bacterial adhesion. In summary, the composite modification can combine the advantages of various modification methods to further improve PEEK’s biocompatibility.

## Blending Modification

Polyether ether ketone is a kind of dental implant material with good physical and chemical properties and elastic modulus close to human bone, which makes it a good application prospect in implant field. However, PEEK’s biological inertia limits its ability to form good bone binding with bone tissue. Therefore, various modification methods have been used to improve its biological activity. In addition to the surface modification mentioned above, blending modification methods are also possible. Blending modification is carried out to prepare composite materials by mixing various biological active substances with PEEK to improve its biological activity. This method can effectively improve PEEK’s biological activities (such as bone formation activity) or antibacterial ability, but mixing other materials into PEEK may have a certain impact on its structure and mechanical properties. Common bioactive substances that can be mixed with PEEK include HA and carbon fiber (CF).

### PEEK/HA Composites

Hydroxyapatite is a bioactive substance with good biocompatibility and bone conductivity, whose ratio of calcium and phosphorus is similar to human bone. It can combine well with bone tissue (Zhang et al., 2019; Zhu et al., 2020; Zhao et al., 2021). Therefore, researchers often mix HA and PEEK to prepare composite material to improve the latter’s biocompatibility. Walsh et al. (2016) prepared an HA and PEEK blend material, then prepared tibial cortical bone defect of sheep and spongy bone defect of tibia and femur, implanted pure PEEK and PEEK/HA composite materials into the defect respectively, and observed the osseointegration by histological detection at 4 and 12 weeks. The results showed that the interface of fibrous tissue was formed around the unmodified sample, while the new bone was formed around the composite and formed bone binding with the surrounding tissue. Then, the authors implanted the composite material into sheep as intervertebral fusion nails and compared it with the allogeneic bone material and pure PEEK material. The results showed that the allograft bone was fractured and reabsorbed, fibrous tissue interfaces were formed around PEEK, and the PEEK/HA composite formed good bone binding at 12 weeks. These results showed that HA significantly improved PEEK’s osteogenic capacity.

The bioactivity of nano hydroxyapatite (N-HA) is better than that of HA alone. Therefore, Zhao et al. (2016) mixed N-HA with PEEK to prepare a composite material with a content of 40% N-HA. Subsequently, human osteoblast-like cells MG-63 were inoculated on the surface of the sample before and after modification to observe the cytocompatibility of the cells before and after modification. It was found that the cell adhesion ability and alkaline phosphatase activity on the surface of n-HA/PEEK composite material were higher than those of the PEEK sample group, but the cell proliferation ability was lower than that of pure PEEK. Proteomics analysis was used to explain that this phenomenon was caused by the increase in the amount of calcium-related proteins and the decrease in the amount of RNA-related proteins in the composite sample group. These results suggested that n-HA can enhance PEEK’s ability to promote cell adhesion and osteogenic differentiation.

Hydroxyapatite is brittle (Ma et al., 2014a), and it always destroy PEEK’s excellent mechanical properties after blending with PEEK. To enhance the mechanical properties of composite materials, Ma J. et al. (2020) used a 3D braiding, self-retention, hot pressing method to prepare an N-HA and PEEK blending material. The nHA/PEEK composites with an nHA content of 6.5 and 14.5 wt% were prepared. *In vitro* characterization tests showed that the hydrophilicity and mineralization of the composites were significantly stronger than those of pure PEEK materials. The mechanical properties of composites were evaluated by a series of mechanical tests. The results showed that owing to the impact of the braided structure, the elastic modulus, hardness, and bending strength of the composite were higher than those of the PEEK sample, and the fracture toughness of the sample with low nHA content was also significantly enhanced. Meanwhile, *in vitro* cytological test results showed that the introduction of nHA effectively improved PEEK’s cell adhesion and proliferation abilities. The above results showed that PEEK’s biological activity could be significantly improved after blending with HA. Although HA is rather brittle and has poor mechanical properties, with the continuous development of research, new preparation methods of mixed materials that can reduce the impact of HA on the mechanical properties of PEEK are being constantly discovered; thus, HA/PEEK composites have a good research prospect.

### PEEK/Carbon Fiber Composites

Carbon fiber has the advantages of high strength and high temperature and corrosion resistance and is usually blended with PEEK to enhance its mechanical properties. Yan M. et al. (2018) prepared CF/PEEK composites by selective laser sintering, and the mechanical results showed that the tensile strength and elastic modulus of PEEK/CF composites were significantly higher than pure PEEK, effectively improving PEEK mechanical properties. Zhao et al. (2017a) prepared PEEK/CF composites by the vacuum hot pressing method and detected the contact angle and friction coefficient of different samples. The results showed that compared with pure PEEK, CF/PEEK’s surface contact angle decreased, hydrophilicity increased, friction coefficient decreased, and wear resistance increased.

To study the specific modification effect of introducing CF into PEEK, Qin et al. (2019) prepared a series of PEEK/CF composites with different CF contents and studied the mechanical properties, thermal properties, and cytotoxicity of the composites. The results showed that compared with pure PEEK, the mechanical properties and thermal properties of CF were significantly enhanced, but they were slightly toxic to cells within a week. The results showed that CF-enhanced PEEK could effectively improve the mechanical properties of PEEK, but the biocompatibility of composite materials was somewhat limited. Therefore, Yan J. H. et al. (2018) introduced a graphene coating on the CF-reinforced PEEK (CFR/PEEK) surface to enhance the biological activity of CFR/PEEK scaffolds. Characterization tests showed that the graphene coating was successfully introduced onto the surface of the CFR/PEEK stent and enhanced the hydrophilicity of the CFR/PEEK material. *In vitro* cytological tests showed that the graphene-coated scaffold surface was more conducive to cell adhesion, proliferation, and osteogenic differentiation, showing good biological activity. In animal experiments, the rabbit femoral condyle defect model was used, and the results of three-dimensional imaging of the formation of new stock around the implant at 4, 8, and 12 weeks as well as histological staining results showed that the graphene-coated implant scaffolds had stronger osteogenic activity.

In addition to graphene, HA was also used to improve the biocompatibility of CF/PEEK composites. Liu et al. (2011) introduced HA which has good biological activity on CF/PEEK and prepared PEEK/HA/CF composites and studied the biocompatibility of the composites by co-culture with osteoblasts *in vitro*. The results showed that the composite was non-toxic to osteoblasts, the cells grew well on the surface of the composite, and the differentiation ability of the surface cells of the composite was improved compared with the control group. These results indicated that the PEEK/HA/CF composite had good cytocompatibility *in vitro*. Furthermore, the results showed that CF could significantly improve PEEK mechanical properties. Although it has limited ability to improve PEEK’s biological activity, other bioactive substances such as HA or graphene can be further introduced to enhance PEEK’s mechanical properties and simultaneously improve its biological activity.

### Other PEEK Composites

In addition to HA and CF, studies have identified a number of bioactive materials for PEEK blends, such as zinc oxide, bioactive glass, titanium dioxide particles, and nano-silicates.

Zinc oxide nanoparticles are multi-functional inorganic nanoparticles with excellent mechanical and biological properties. Studies showed that ZnO exhibits significant antibacterial activity (Yamamoto, 2001). Diez-Pascual and Diez-Vicente (2014) and Diez-Pascual et al. (2014) first modified the surface of ZnO nanoparticles, and then prepared PEEK/ZnO nanocomposites to study the mechanical and antibacterial properties of composites. The results show that PEEK/ZnO nanocomposites prepared by modified nano-ZnO have better mechanical properties than those prepared by unmodified nano-ZnO., but PEEK/ZnO nanocomposites had good antibacterial properties against *E. coli* and *S. aureus* regardless of whether ZnO was modified. The antibacterial property enhanced with the increase in the ZnO content, and the best antibacterial property was obtained at 7.5 wt% of the modified nanoparticles. Hao et al. (2018) synthesized PEEK/ZnO nanocomposites by conventional melting blending and compression molding and studied the properties of the composites. The results showed that the mechanical properties and thermal stability of the composites were significantly improved and the composites had good cellular compatibility.

In addition to ZnO, TiO_2_, Al_2_O_3_, MoS_2_, nanometer calcium silicate (n-CS), and other active substances were also used to prepare mixed materials with PEEK to improve its biological and mechanical properties. Wu et al. (2012) prepared PEEK/TiO_2_ composite material and through biological detection, found that its cellular compatibility was enhanced compared with pure PEEK; moreover, the osteoblasts were more likely to stick to the exposed position of TiO_2_ particles. Kuo et al. (2005) prepared PEEK/SiO_2_ and PEEK/Al_2_O_3_ composites with nanometer SiO_2_ and Al_2_O_3_ particles respectively, and studied the mechanical properties of the composites. The results showed that when the introduced particle mass fraction was 5.0–7.5%, the hardness, elastic modulus, and tensile strength of the composites could be significantly improved. Lv et al. (2020) mixed molybdenum disulfide (MoS_2_, MS) nanosheets with PEEK to prepare MS/PEEK composites with an MS content of 4 and 8 wt%, and studied the tribological and mechanical properties and cellular compatibility of the composites. The results showed that the surface of the composite became rougher and hydrophilic, the mechanical properties were enhanced, and the modification effect improved with increasing MS content. Cytological tests showed that the growth of rat bone marrow mesenchymal cells on the surface of composite material was better than that of the pure PEEK sample group, indicating that MS could effectively improve PEEK’s mechanical properties and cellular compatibility. Ma et al. (2014b) prepared n-CS/PEEK bioactive composite by compound injection molding method. The mechanical properties, hydrophilicity, bioactivity, and cytocompatibility of the composites were studied. The results showed that compared with pure PEEK, the mechanical properties, hydrophilicity, and mineralization ability of the composite were significantly improved. The results of cell experiments showed that n-CS/PEEK composites significantly promoted the ability of cell attachment, proliferation, diffusion, and osteogenic differentiation.

These results indicate that various blend modifications can enhance PEEK biological activity by introducing active substances or changing PEEK surface morphology, but most of the studies have been limited to *in vitro* experiments, and further *in vivo* and clinical trials are needed to verify the modification effect.

## Conclusion

Polyether ether ketone and its composites play an important role in the field of oral repair given their excellent physical and chemical properties as well as biological properties. Its machinability enables PEEK to accurately manufacture various kinds of implants with complex structures. Its excellent properties such as stable chemical properties, good biosecurity, and elastic modulus are close to human dense bone and therefore make it an excellent oral implant potential material. The biological modification methods described above are all aimed at improving PEEK’s biological activity. Each modification method has its advantages and disadvantages. Despite considerable experimental studies, modification technologies are still immature and lack sufficient clinical data to prove the clinical efficacy of these modification methods. Therefore, future research should focus on the development of more efficient and practical modification methods and clinical practice, and the exploration of PEEK modification methods to address different branches of stomatology.

## Author Contributions

All authors listed have made a substantial, direct and intellectual contribution to the work, and approved it for publication.

## Conflict of Interest

The authors declare that the research was conducted in the absence of any commercial or financial relationships that could be construed as a potential conflict of interest.
